# Simulating Facebook Advertisements to Establish Cost per New HIV Diagnosis Using Routine and Targeted Models in a Local Population

**DOI:** 10.3390/healthcare10071195

**Published:** 2022-06-26

**Authors:** John J. Hanna, Ank E. Nijhawan, Christoph U. Lehmann, Richard J. Medford

**Affiliations:** 1 Division of Infectious Diseases and Geographic Medicine, University of Texas Southwestern, Dallas, TX 75390, USA; ank.nijhawan@utsouthwestern.edu (A.E.N.); richard.medford@utsouthwestern.edu (R.J.M.); 2 Clinical Informatics Center, University of Texas Southwestern, Dallas, TX 75390, USA; christoph.lehmann@utsouthwestern.edu; 3 Department of Population and Data Sciences, University of Texas Southwestern, Dallas, TX 75390, USA; 4 Department of Bioinformatics, University of Texas Southwestern, Dallas, TX 75390, USA; 5 Department of Pediatrics, University of Texas Southwestern, Dallas, TX 75390, USA

**Keywords:** Facebook advertisements, personalized advertisements, social media, precision medicine, public health informatics, public health communications, consumer health informatics, population health, human immunodeficiency virus, diagnosis, acquired immunodeficiency syndrome

## Abstract

Background: Undiagnosed human immunodeficiency virus (HIV) infection remains a public health challenge. We explore Facebook (FB) advertisement (Ads) cost per new HIV diagnosis using non-targeted Ads, a routine testing model against targeted Ads, and a focused testing model in Texas. Methods: On 14 October 2021, we created (without launching) Texas-based, USD 10 targeted (using criteria matching HIV populations at risk) and non-targeted FB Ads for 10 days. In the process of creating the Ads, we collected estimated audience size, daily reach, and daily clicks. We estimated Ad cost for each new HIV diagnosis for targeted and non-targeted Ads using new HIV diagnosis rates from focused and routine testing campaigns. Results: The Ad costs per new HIV diagnosis from the targeted model were 4.74, 2.86, 5.28, and 2.88 times lower for men, Black, Hispanic, and all age groups, respectively, when compared to the non-targeted model. The wider the gap was between new HIV diagnosis rates in a population for focused and routine testing, the more cost-effective targeted Ads became. Conclusions: Among HIV populations at risk, targeted FB Ads are more cost-effective for detecting new HIV infections than non-targeted Ads. This cost-effectiveness increases in locations where focused testing increases new HIV diagnosis rates, compared to routine testing.

## 1. Introduction

Human immunodeficiency virus (HIV) morbidity and mortality affect the United States of America (USA) regions disproportionately [1]. Southern states have the highest rates of new HIV diagnoses (estimated to constitute 51% of new HIV cases annually, nationally) and, subsequently, have a larger, more geographically dispersed population of people living with HIV (PLWH) [1]. The high rates of HIV incidence and prevalence in the South are accompanied by high rates of social determinants of health (SDOH) risk indicators, including lack of insurance, vacant housing, household incomes below the federal poverty level, and rural populations [2]. These observations warrant new initiatives to reduce HIV infections in the South [3].

Texas has one of the highest rates of new HIV diagnoses among the Southern states, with 18.2 new cases per 100,000 people. Texas also has a proportionally lower estimated rate of pre-exposure prophylaxis (PrEP) coverage compared to other states. Including the undiagnosed HIV population, Texas has the highest estimated incidence rate of HIV nationally, with 4500 new cases and a prevalence rate of 113,300 HIV cases per 29 million as of 2019 (1 out of every 256 Texans) [2]. With a rate of 18.4%, the state of Texas also has the highest burden of uninsured persons nationally and one of the highest rates of households living below the federal poverty level (10.5%) [2].

The burden of HIV and associated SDOH are not only disproportionately distributed regionally, but are also more likely to affect certain race/ethnicity groups and different age groups. In the South, Black/African Americans and people 25–34 years old have the highest rates of new HIV diagnoses, regardless of urban or non-urban environments [4]. In Texas in 2018, the highest number of new HIV diagnoses were among Hispanic males (42% of all HIV diagnoses + males) and Black females (51% of all HIV diagnoses + females). While the age group of 25–34 years had the highest rates of new diagnoses, women tended to be diagnosed at a later age than males. Among Texas metropolitan areas, Dallas eligible metropolitan area (EMA) (25%) and Houston EMA (31%) accounted for 56% of Texas PLWH in 2018 [5].

New, innovative public health approaches are needed to reach populations at risk for HIV to increase testing and reduce the incidence of undiagnosed and untreated HIV. In the last decade, social media advertisements (Ads) have enabled health organizations to identify populations with characteristics that match risk factors for diseases [6,7,8] and to reach populations at risk for research recruitment [9,10,11,12,13,14,15]. Among social media platforms, Meta Inc., (Menlo Park, CA, USA) continues to lead the market, with 2.91 billion active users monthly in the fourth quarter of 2021 [16]. The platform’s high penetration and the wide array of permitted detailed targeting criteria allow researchers to use Facebook (FB) Ads to recruit difficult-to-reach populations [17,18,19].

Data from a randomized control trial of HIV and substance use interventions showed that gender minority adolescents and young adults were easier to recruit on social media platforms compared to in-person for HIV interventions [20]. Recent international studies that used social media to promote sexual health resulted in increased HIV testing and linkage to care in high-risk and difficult-to-reach young men who have sex with men (MSM) [21,22]. Similarly, the Keeping it LITE study and the START study were nationally successful in recruiting gender minority participants effectively and efficiently from social media Ads [23,24]. Compared to other social media platforms, FB Ads for HIV prevention have yielded the lowest cost per eligible contact for young MSM, and Instagram Ads have yielded the highest proportion of eligible contacts who were racial or ethnic minorities [25].

In this study, we aim to evaluate the feasibility of FB Ads to reach MSM at high risk for HIV infection in Texas. We compare the cost and efficacy between targeted FB Ads followed by the focused HIV testing of individuals at the highest risk of HIV acquisition (targeted model) against non-targeted FB Ads followed by routine HIV testing (non-targeted model). To estimate the cost for each new HIV diagnosis in both models, we use the FB platform Ad estimates, the healthcare industry average conversion rate on FB (percentage of visitors to a website that do what the advertiser wants them to do, e.g., get tested) [26], and the rates of new HIV diagnoses from focused and routine testing [5].

## 2. Materials and Methods

FB provides estimates for Ad reach and Ad clicks based on adjusted Ad budgets even prior to running the Ads. On 14 October 2021, we created (without actually launching) 10-day, Texas-based, USD 10 targeted and non-targeted FB Ads for different age groups in Texas counties with high HIV prevalence. Ads placements included FB, FB Messenger, and Instagram. For the targeted FB Ads group, we used the following FB criteria to match the MSM population at highest risk for HIV: men with FB-defined interests in LGBT culture, LGBT community, homosexuality, same-sex marriage, and same-sex relationships.

We attempted to target the age group category at highest risk in the 2018 Texas HIV epidemiologic profile. However, we had to adjust the age group from 15–24 to 18–24, as FB does not allow the granular targeting of users younger than 18 years. Similarly, FB does not allow race/ethnicity-based targeting, and we used interests in African American culture and Hispanic cultures as a proxy for the African American and Hispanic populations. For transgender women and men, we used their interest in transgender issues and their corresponding gender for our FB targeting criteria.

For each targeted group/subgroup, we collected the FB-provided estimated audience size, estimated daily reach, and estimated daily clicks per Ad. We then estimated the average Ad cost for each new HIV diagnosis for targeted and non-targeted Ads. We leveraged new HIV diagnosis rates from focused testing and routine testing campaigns in Texas based on the following formula:

Estimated Ad cost per new diagnosis = daily Ad cost/(estimated daily clicks × average healthcare conversion rate × average rate of new diagnosis)

Using a USD 10 budget for a 10-day-long Ad translated into a daily Ad cost of USD 1. We used the daily cost to compare the FB-provided estimated daily reach and estimated daily clicks for all age groups. We used the average healthcare industry conversion rate on Facebook to estimate the percentage of Facebook users who would convert as a result of the Ads, which in this case translated to undergoing HIV testing. Based on subgroups, we estimated the rates of new HIV diagnoses based on the 2018 Texas HIV epidemiologic profile. For the targeted model, we used the rates of new HIV diagnoses from focused testing (testing a population that is more at risk for HIV). For the non-targeted model, we used HIV new diagnosis rates from routine testing (testing patients independent of their HIV risk).

## 3. Results

On 14 October 2021, the estimated audience size for the model targeting MSM in Texas included 1,350,000 individuals compared with an estimated 11,050,000 Texan men in the non-targeted model. The cost for each new HIV diagnosis was 4.7 times lower for the targeted model (targeting MSM in Texas: USD 33.06) compared with the non-targeted model (targeting all Texan men: USD 156.74). 

**Figure 1:**
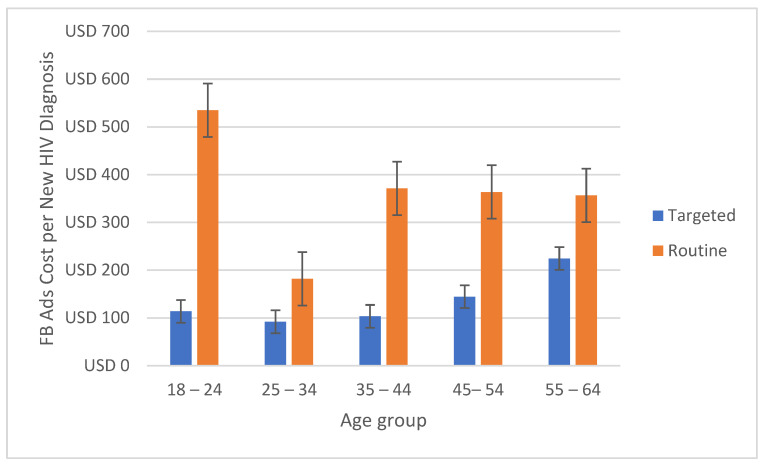
Estimated Ad cost per new HIV diagnosis comparison between targeted and non-targeted models for age groups in Texas, USA.

**Figure 2:**
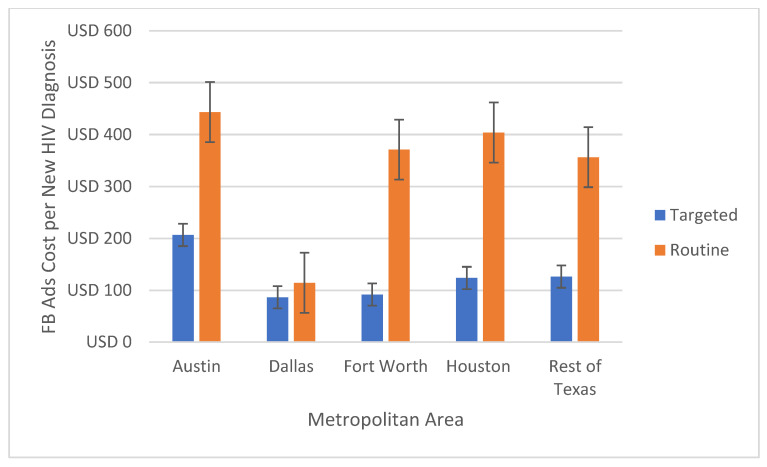
Estimated Ad cost per new HIV diagnosis comparison between targeted and non-targeted models for Texan metropolitan areas in Texas, USA.

**Figure 3:**
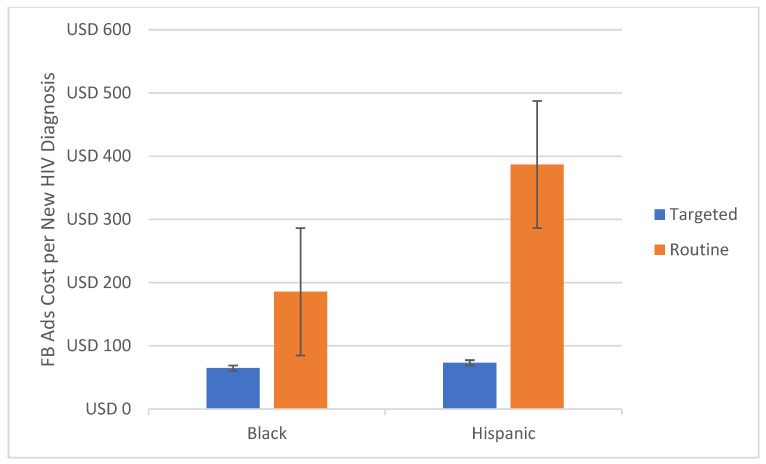
Estimated Ad cost per new HIV diagnosis comparison between targeted and non-targeted models for race/ethnicity in Texas, USA.

The average Ad cost per new HIV diagnosis was lower in the targeted model for all subgroups (Table 1). The costs for targeted campaigns were half in all age groups (Figure 1) and 2.4 times lower in the main affected Texan metropolitan areas (Figure 2). In Hispanic and Black/African American men, the targeted model’s costs for each new HIV diagnosis were 5.3 and 2.9 times lower, respectively (Figure 3). 

Among the Texas metropolitan areas, Dallas had the highest rate of new HIV diagnoses through focused and routine testing. Dallas also had the lowest estimated cost for each new HIV diagnosis for targeted and non-targeted Ads. Austin had the lowest rates for new HIV diagnoses and the highest cost for new HIV diagnoses (targeted or not). The ratio of costs for new diagnoses for the targeted and the non-targeted model was lowest in Dallas, which also had the lowest ratio between new diagnosis rates from focused vs routine testing (1/0.3). The cost ratio was highest in Fort Worth, which also had the highest ratio between new diagnosis rates from focused vs routine testing (0.9/0.1).

## 4. Discussion

The “Ending the HIV Epidemic: A plan for America” initiative, empowered by the U.S. Department of Health and Human Services (HHS), has launched work with communities, aiming to leverage critical scientific advances in HIV prevention, diagnosis, treatment, and outbreak response and redirect resources to areas where HIV transmission occurs most frequently. The initiative has the goal to reach a 75% reduction in new HIV infections by 2025 and at least a 90% reduction by 2030 [27].

To explore the most cost-effective means of reducing HIV infections, we compared FB Ads’ cost per new HIV diagnosis from targeted and non-targeted models in five Texas counties (Bexar, Dallas, Harris, Tarrant, Travis) from the targeted 57 priority jurisdictions in the cross-agency HHS initiative. In all Texas areas, age groups, and race/ethnicity (Table 1), Ad cost per new HIV diagnosis was lower in the targeted model (targeted Ads followed by focused HIV testing) than the non-targeted model (non-targeted Ads followed by routine HIV testing).

The targeted model’s lower Ad cost per new HIV diagnosis was in part caused by the higher new HIV diagnosis rates from focused HIV testing. FB Ad cost also varied among each targeted population, as FB uses an Ad auction to determine the best Ad to show to a person at a given point in time [28]. The FB Ad auction is designed to show the Ad with the highest total value to FB users. The Ad total value is based on three major factors: Ad bid, estimated action rates, and Ad quality. The three variables that determine Ad total value, along with seasonality, are the main factors that affect the cost of FB Ads at any point in time. In our study, we collected data on the same day using the same video Ad design and budget to control seasonality and Ad quality among all groups. However, the two other variables that contribute to FB Ads’ total value are audience specific: the Ad bid compared to other Ads targeting the same audience, and the estimated action rates based on the FB-estimated probability that showing an Ad to a person leads to the desired outcome of the advertiser.

The results from our study also cast light on the effect of different rates of new HIV diagnoses from routine and focused testing in a certain population on the cost of FB Ads. We observed that the wider the gap in a population between new diagnosis rates from focused HIV testing and new diagnosis rates from routine testing, the more cost-effective targeted FB Ads become in the same population. This finding can guide the allocation of resources based on focused/routine testing rates and can be used to prioritize reach models for each population at risk for HIV.

We focused on populations at risk for HIV in Texas to explore feasibility. However, considering the input variables, this strategy can be applied to any population at risk for a given disease if a population can be represented through a set of targeting criteria/interests on FB Ads and public action rate data are available. In 2021, An et al. [15] proposed a novel precision public health campaign framework to structure and standardize the process of designing and delivering tailored health messages to target population segments on social media, demonstrating their framework through two case studies: breast cancer screening in Qatar, and public health campaigns for promoting flu vaccination in Qatar. In the proposed framework, defining priority audience and evaluation metrics are key factors of the first stages of a social media public health campaign. Our MSM/HIV at-risk audience definitions on FB, FB Messenger, and Instagram, along with our model characteristics, can help public health communities in these first stages of building a public health campaign framework to tackle the HIV epidemic.

Other social media platforms including Twitter, LinkedIn, Snapchat, and TikTok also allow promoters to use basic demographic traits for targeting their audience; however, these rich demographic traits are only available on FB and Snapchat [15]. Platform penetration in a specific population’s demographic also plays a key role in selecting the best platform. For example, considering the highest rates of undiagnosed HIV are among the 13–24 age group, platforms such as TikTok or Snapchat that have high penetration in this demographic should also be considered. 

While targeted Ads on social media can be effective and cost efficient for HIV prevention, there exists an ethical concern of privacy when targeting vulnerable populations (e.g., Blacks, Hispanics, LGBTQ), whereby an identifiable digital trail is maintained [29,30]. It is thus imperative for researchers and public health officials to act as stewards of this potential untapped resource and to advocate for these vulnerable populations when using these platforms. Additionally, other platforms, including Google and TikTok, have measures in place to protect the privacy and safety of teens. While these measures limit targeting adolescents (where underdiagnosed HIV is highly prevalent), it is important to create collaborative initiatives between public health communities and social media platforms, similar to the COVID-19 response, to aid in ending the HIV epidemic in digital America by 2030.

## 5. Conclusions

Targeted FB Ads are more cost-effective than non-targeted Ads among HIV populations at risk, across all age groups, and in locations where focused testing yields substantially higher new HIV diagnosis rates compared with routine testing. Our study results can guide public health agencies and local communities in optimizing their resource utilization to address the HIV epidemic, as social media Ad strategies are useful for improving HIV prevention, testing, and treatment. Our future efforts will aim to compare FB Ads’ outcomes from launched FB Ad campaigns, including social media Ad metrics, conversion rates, and cost per action among different FB Ad targeting strategies based on FB-defined interests and locations targeting MSM populations at risk for HIV locally in Dallas, TX, in collaboration with a local community initiative.

## 6. Limitations

Our study has several limitations that must be acknowledged. Firstly, our proposed model is subject to the limitations of the social media platform and will continue to evolve while the platform tools evolve. Our modeling was based heavily on estimates provided by the FB Ads platform without running Ads. The audience size and reach estimates were provided by FB algorithms based on active FB and Instagram users which we were unable to validate. The adjusted targeting criteria were not designed to match a census population. The Ads’ estimated daily reach and daily clicks were also subject to the platform algorithms and could vary based on Ad auction factors, as explained in-detail in the discussion section of this study.

Secondly, the conversion rate may also vary based on the Ad quality and its appropriateness to the viewing users. In our model, we used the average healthcare conversion rate on FB by WordStream based on a sample of 256 of their US-based clients in all industries who were advertising on FB between November 2016 and January 2017 [26]. Our study also did not examine the effect of different Ad designs that could variably affect the Ad outcomes, as this would have required user interactions on launched Ads for optimum testing.

## Figures and Tables

**Table 1:** Average Ads cost per new HIV diagnosis among targeted and non-targeted models in Texas, USA.

	Targeted Ads Followed by Focused Testing (Targeted Model)			Non-Targeted Ads Followed by Routine Testing (Non-targeted Model)		
	New Diagnosis Rate	Average Estimated Audience Size on Facebook	Average Cost Per New HIV Diagnosis	New Diagnosis Rate	Average Estimated Audience Size on Facebook	Average Cost Per New HIV Diagnosis
Men	1.0%	1,350,000	USD 33.06	0.2%	11,050,000	USD 156.74
Black	0.8%	289,550	USD 64.94	0.2%	2,100,000	USD 185.53
Hispanic	0.8%	784,300	USD 73.31	0.1%	3,050,000	USD 386.85
18–24	0.8%	326,450	USD 113.64	0.1%	4,800,000	USD 534.76
25–34	0.9%	483,300	USD 91.83	0.2%	6,900,000	USD 181.82
35–44	0.8%	236,250	USD 103.31	0.1%	4,900,000	USD 371.06
45–54	0.6%	125,000	USD 144.30	0.1%	3,400,000	USD 363.64
55–64	0.3%	76,700	USD 224.47	0.1%	2,350,000	USD 356.51
65+	0.2%	60,000	USD 413.22	0.0%	2,050,000	
Austin	0.4%	110,100	USD 206.61	0.1%	1,750,000	USD 443.46
Dallas	1.0%	264,700	USD 86.58	0.3%	4,650,000	USD 114.35
Fort Worth	0.9%	162,000	USD 91.83	0.1%	3,100,000	USD 371.06
Houston	0.7%	288,500	USD 123.69	0.1%	4,750,000	USD 404.04
San Antonio	0.6%	121,400	USD 168.35		2,050,000	
Rest of Texas	0.8%	189,150	USD 126.26	0.1%	9,700,000	USD 356.51

## Data Availability

Project data available at https://github.com/johnjero/OFBADSHIV.
